# Prevention of Meningococcal Disease: Knowledge, Attitudes, and Practices of General Practitioners and Primary Care Pediatricians in South Italy

**DOI:** 10.3390/vaccines12080889

**Published:** 2024-08-06

**Authors:** Silvia Angelillo, Concetta Paola Pelullo, Francesca Licata, Raffaele Lanzano, Francesco Napolitano, Gabriella Di Giuseppe

**Affiliations:** 1 Department of Health Sciences, University of Catanzaro “Magna Græcia”, 88100 Catanzaro, Italy; 2 Department of Medical, Human Movement and Well-Being Sciences, University of Naples “Parthenope”, 80133 Naples, Italy; 3 Department of Experimental Medicine, University of Campania “Luigi Vanvitelli”, 80138 Naples, Italy

**Keywords:** general practitioners, meningococcal disease, primary care pediatricians, practices, prevention, vaccination

## Abstract

Background: The purpose of this study was to evaluate the knowledge, attitude, and current practices about prevention of meningococcal disease among general practitioners (GPs) and primary care pediatricians (PCPs) in Italy. Methods: A cross-sectional survey was carried out between February 2022 and July 2023 among a random sample of GPs and PCPs in Southern Italy. The data were collected using a questionnaire accessible via an internet link with the free software Google Forms^®^. Results: Regarding the participants’ knowledge toward meningococcal vaccinations, 84.2% of the PCPs and more than half of the GPs (55.2%) knew that the meningococcal B (MenB) vaccination is recommended for infants from the second month of life and 84.2% and 82.7% of the PCPs were aware that quadrivalent meningococcal ACWY (MenACWY) vaccine is recommended for children in the second year of life and adolescents, respectively. The GPs and PCPs considered vaccination against meningococcal disease to be very effective and safe with average values of 8.8 and 8.7, respectively, on a scale ranging from 1 to 10. Those with an older age, those who knew the medical conditions that expose patients to a higher risk of contracting meningococcal disease, and those who self-rated their knowledge on meningococcal disease as excellent/very good were more likely to consider the vaccination to be very effective and safe. Only 15.5% of the GPs and more than half of the PCPs (54.3%) administered anti-meningococcal vaccines to their patients. GPs and females were less likely to administer anti-meningococcal vaccines to their patients, whereas those who acquired information on meningococcal vaccinations by scientific journals were more likely to administer meningococcal vaccines. Conclusions: The findings of the survey highlighted the need of a greater engagement of GPs and PCPs in the immunization campaigns in order to increase meningococcal vaccination coverage.

## 1. Introduction

*Neisseria meningitidis*, with serotypes A, B, C, W, X, and Y, is responsible for most meningococcal diseases globally among people of all ages. Although over time there have been improvements in therapies for the disease, approximately 10% to 15% of patients die, and about 20% of survivors may have permanent sequelae [1]. In 2018, among 30 European countries, 3.316 confirmed cases of meningococcal disease were reported [2], and, in Italy, 57 cases of invasive meningococcal disease were reported in 2022, whereas in 2021 and 2020, there were 25 and 74 cases, respectively [3]. The incidence was 0.12 cases/100,000 and 0.04 cases/100,000 inhabitants in 2020 and in 2021, respectively, with an increase to 0.1 cases/100,000 inhabitants in 2022 [3,4]. The most recent data show that serogroup B was found to be responsible for all cases in 2021 in children under one year of age and it has also increased in the young aged 15–24 years [4]. Although meningococcal disease is more frequent in children and young adults, it is possible that it occurs among subjects of any age with higher risk due to underlying medical conditions, such as thalassemia, diabetes, serious chronic liver diseases, congenital or acquired immunodeficiencies, and asplenia. Therefore, vaccination remains the most effective instrument to prevent the different types of meningococcal disease. The Italian vaccination prevention plan provides routine immunization with quadrivalent meningococcal ACWY (MenACWY) and meningococcal B (MenB) for children or adolescents and for patients with thalassemia, sickle cell anemia, immune disorders, type 1 diabetes, chronic kidney disease, congenital immunodeficiencies, HIV infection, chronic liver disease, HPV infection, and asplenia and not specifically for older adults [5]. 

The data on vaccination coverage for 2021 show that for the cohort of children born in 2019, coverage at 24 months was 79.7%, 73.4%, and 54.2% for MenB, meningococcal C (MenC), and MenACWY, respectively, while for adolescents born in 2005, the 16-year coverage was 58.9% for MenC and 58.5% for MenACWY [6]. However, the vaccination coverage in both cohorts is still quite low despite the information campaigns on the importance and usefulness of these vaccinations.

The topic of communication on vaccination against meningococcal disease is part of the broader debate on communication relating vaccination strategies to health promotion and on the importance of effective communication to provide correct, complete, and understandable information, which can counter the spread of false information, not based on scientific evidence, and which can facilitate individuals in making free and informed choices. Therefore, the knowledge and attitudes of healthcare workers (HCWs), in particular general practitioners (GPs) for the adult population and primary care pediatricians (PCPs) for parents of children and adolescents, are important factors that can influence the acceptability of vaccinations, considering that Gps and PCPs play a key role in the dissemination of correct information on the importance of vaccinations. A widespread acceptability of these vaccinations in the target populations will also depend on their knowledge, attitudes, and practices. While there are several studies that have investigated these aspects for other vaccinations [7,8,9,10], very limited research has been dedicated to the prevention of meningococcal disease. Therefore, the purpose of this study was to evaluate the knowledge, attitude, and current practices about meningococcal vaccination among GPs and PCPs in Italy.

## 2. Materials and Methods

### 2.1. Design and Sample

This study is part of a larger research project on the prevention of meningococcal disease in different groups of individuals [11] and it was carried out between February 2022 and July 2023. The target population consisted of GPs and PCPs of the provinces of Naples and Caserta (Campania region) and Catanzaro and Cosenza (Calabria region), located in Southern Italy, and the participants of the survey were randomly selected among the GPs and PCPs of the Local Health Units (LHUs). In particular, a random sample of LHUs was selected across the Campania and Calabria regions and a random sample of 630 PCPs were recruited from the list provided by each selected LHU.

A minimum sample size of 480 was calculated assuming that 75% of the physicians recommended the vaccinations against meningococcal disease according with the findings of previous investigations [12,13,14], a confidence interval of 95%, a margin of error of 5%, and a response rate of 60%. 

### 2.2. Data Collection Procedure and Survey Instrument

The randomly selected GPs and PCPs were invited via e-mail to complete a questionnaire accessible via an internet link using the free software Google Forms^®^ that allowed for an anonymous response, once from each unique IP address to ensure single entries. The first page of the questionnaire included the online informed consent statement, and only if the participants provided their consent they were allowed to proceed to the next page. Moreover, the following information was reported on this page: a brief description of the study objectives, the survey team’s contact details, information on the voluntary participation and the confidentiality and anonymization of the responses, that they do not have the option to skip or leave question blank or to choose non-responses, that they were allowed to withdraw from the survey at any point, and that no compensation or other incentives were offered. The questionnaire was pre-tested among a convenience sample of 20 GPs, excluded from the final analysis, for clarity, consistency, and comprehensibility, with some minor modifications based on the feedback received.

The questionnaire, which needed approximately 10 min to be completed, consisted of 31 items grouped into five sections (Supplementary File: Questionnaire). The first section included questions on the participants’ sociodemographic (gender, age, marital status, and number of children) and professional characteristics (year of graduation, role, number of hours worked per week, and number and age of patients seen in a week). The second section measured the knowledge about meningococcal disease by 9 statements regarding incidence, lethality, risk groups, and vaccination. The answers for the different items had the option for “yes”, “no”, and “do not know” or the selection from a list that allowed for single or multiple responses. The third section evaluated the risk perception of getting the disease for their patients; the concern about the safety, side effects, and effectiveness of the meningococcal vaccines; and the role of the HCWs in encouraging patients to be vaccinated against meningococcal diseases. Four questions were on a 10-point Likert-type scale ranging from 1 (to a very small attitude) to 10 (to a very large attitude) and one questions on a 5-point Likert-type scale, and the response options were strongly disagree, disagree, uncertain, agree, and strongly agree. The fourth section assessed the practices toward the meningococcal vaccine by asking six questions regarding the frequency of verifying the patients’ immunization status, the recommendation of the vaccine in the different age groups, the administration of the vaccine, and the patients’ acceptability. These questions had a “yes”, “no”, and “do not know” format and open-ended responses. The last section included questions regarding the information sources about meningococcal vaccination, the level of their satisfaction with the information received, and the needs of additional information on this topic.

The study protocol and the questionnaire were approved by the Ethics Committee of the Teaching Hospital of the University of Campania ‘‘Luigi Vanvitelli” (N. 0008660/I, 15 April 2020).

### 2.3. Statistical Analysis

The statistical analysis was performed using the software Stata Version 18 [15]. Firstly, a descriptive analysis was performed, and the results are reported as frequencies and percentages. Secondly, chi-square and Student’s *t*-test were performed for the bivariate analysis to assess the association between each independent variable and the outcomes of interest. Moreover, two multivariable logistic models using a stepwise procedure were built to assess the independent predictors of the following outcomes of interest: GPs and PCPs who considered vaccination against meningococcal disease to be very effective and safe (no = 0; yes = 1) (Model 1) and physicians who administered vaccines against meningococcal disease to their patients (no = 0; yes = 1) (Model 2). The independent variables with a *p*-value < 0.25 in the bivariate analysis were included in the final models according to Hosmer and Lemeshow’s model-building strategy [16], and values of *p* = 0.2 and *p* = 0.4 were used to select candidate variables for inclusion and exclusion in the models. The following variables were included in all the models: gender (male = 0; female = 1), age (continuous, in years), profession (general practitioners = 0; primary care pediatricians = 1), marital status (others = 0; married/cohabitant = 1), length of professional practice (continuous, in years), hours worked per week (continuous), those who self-rated their knowledge on meningococcal disease as excellent/very good (no = 0; yes = 1), those who knew the medical conditions that expose patients to a high risk of contracting meningococcal disease (no = 0; yes = 1), those who need additional information on meningococcal disease and related vaccinations (no = 0; yes = 1), and scientific journals as the main source of information on meningococcal vaccinations (no = 0; yes = 1). Moreover, those who verified the patients’ immunization status during their professional practice (no = 0; yes = 1) and those who recommend the meningococcal vaccines to their patients (no = 0; yes = 1) were included in Model 2. The results of the logistic regression models were measured using Odds Ratios (ORs) with their 95% confidence intervals (CIs) and two-tailed tests were used, and a *p*-value ≤ 0.05 was considered statistically significant.

## 3. Results

### 3.1. Sociodemographic and Professional Characteristics of Participants

Of the 630 selected participants, 507 agreed to participate and fill out the online questionnaire for a response rate of 80.5%. In Table 1, the main sociodemographic and professional characteristics of the participants are summarized. Just over half of the sample was females (50.9%), the mean age was 51.6 years with a range of 28 to 73 years, and more than two-thirds were married/cohabiting (77.5%) and have at least one child (67.2%). Moreover, 68.1% were GPs, the mean length of professional practice was 23 years, and the participants reported working an average of 36.7 h per week.

### 3.2. Knowledges

A large majority (86.6% of the PCPs; 79.2% of the GPs) were aware that in Italy the incidence of meningococcal disease is low, more than half (64.1% of the PCPs; 60.2% of the GPs) knew that the lethality rate ranges from 5% to 10%, and more than two-thirds of the participants (90.7% of the PCPs; 83.5% of the GPs) correctly identified *Neisseria meningitidis* serotype B among the most frequent serogroups responsible for meningococcal disease in Italy, whereas a lower proportion of the participants indicated serotype C (68.9% of the PCPs; 51.5% of the GPs), serotype W135 (62.2% of the PCPs; 48.5% of the GPs), serotype A (27.1% of the PCPs; 33.1% of the GPs), and serotype Y (24.5% of the PCPs; 19.3% of the GPs). When asked about the age and conditions that expose the patients to a higher risk of contracting meningococcal disease, 91.6% of the PCPs and 76.2% of the GPs reported that subjects aged < 5 were at higher risk of contracting meningococcal disease, whereas a lower proportion of the participants (19.6% of the PCPs; 35.8% of the GPs) reported that subjects aged ≥ 65 years were at higher risk. Moreover, a large majority (87.1% of the PCPs; 79.9% of the GPs) knew the medical conditions that expose patients to a higher risk of contracting meningococcal disease. Regarding the participants’ knowledge toward meningococcal vaccinations, 84.2% of the PCPs and more than half of the GPs (55.2%) knew that the MenB vaccination is recommended for infants from the second month of life, and 84.2% and 82.7% of the PCPs were aware that MenACWY vaccine is recommended for children in the second year of life and adolescents, respectively. Moreover, approximately two-thirds of the GPs (64.4%) were aware that MenACWY vaccine is recommended for adolescents. Overall, almost half of the PCPs self-rated as excellent/very good their knowledge on meningococcal disease (47.7%) and the related vaccination strategy (49%), while less than one in five GPs self-rated their knowledge as excellent/very good (19.5%). 

### 3.3. Attitudes

The GPs and PCPs considered it unlikely that their patients would get meningococcal disease with a mean value of 3.3 on a scale ranging from 1 to 10. A positive attitude toward immunization against meningococcal disease was found in the participants. Indeed, the GPs and PCPs considered vaccinations against meningococcal disease to be very effective and safe with average values, respectively, of 8.8 and 8.7, on a scale ranging from 1 to 10, and only 15.4% of the PCPs and 13.6% of the GPs believe it is probable that their patients can experience a serious side effect following the administration of anti-meningococcal vaccines. The results of the multivariate logistic regression analysis showed that those with an older age, those who knew the medical conditions that expose patients to a higher risk of contracting meningococcal disease, and those who self-rated their knowledge on meningococcal disease as excellent/very good were more likely to consider vaccinations against meningococcal disease to be very effective and safe (Table 2). Moreover, almost all the respondents (92.8% of the PCPs; 91.5% of the GPs) agreed or strongly agreed that physicians should promote adherence to the recommended vaccinations against meningococcal disease even in hesitant patients.

### 3.4. Practices

A large majority of the respondents recommended meningococcal vaccines to children (98.7% of the PCPs; 61.1% of the GPs), adolescents (97.3% of the PCPs; 77.9% of the GPs), adults (79% of the GPs), and patients at high risk of contracting meningococcal disease due to their medical conditions (78.8% of the PCPs; 83.4% of the GPs), and two-thirds (68.1% of the PCPs; 67.1% of the GPs) reported that during their professional practice they verified the patients’ immunization status. Moreover, approximately one-third of the respondents (33.1% of the PCPs; 27.5% of the GPs) considered the acceptability of meningococcal vaccinations by parents or their patients as very high or high. Only 15.5% of the GPs and more than half of the PCPs (54.3%) administered vaccines against meningococcal disease to their patients and the most frequently reported reasons for not administering vaccines were that it is not a skill of GPs and PCPs (42.4%), there is a lack of time (32.3%), and that anti-meningococcal vaccines are not provided to GPs and PCPs by the vaccination ambulatory centers (13.4%). The results of the multivariate logistic regression analysis showed that GPs and females were less likely to administer anti-meningococcal vaccines to their patients, whereas those who acquired information on meningococcal vaccinations by scientific journals were more likely to administer anti-meningococcal vaccines (Table 2). 

### 3.5. Sources of Information

Scientific journals were the main source of information on vaccinations (64.7%) followed by the internet (39.4%), mass media (29.3%), continuing medical education courses (23.2%), colleagues (16.3%), and professional associations (9.2%). Moreover, only 15.4% of the participants participated in courses/scientific conferences on meningococcal disease and the related vaccination strategies in the last 12 months, 22.4% believed that the information received about the prevention of meningococcal disease was very useful with a mean value of 7.9 on a scale ranging from 1 to 10, and 87.4% reported the need for additional information on meningococcal disease and the related vaccinations.

## 4. Discussion

The study results provide interesting information that can be used in the vaccination strategies against meningococcal disease in order to improve the coverage in at-risk patients. 

It is important to consider that the comparisons with previous investigations conducted in other countries may be difficult due to differences in the characteristics of the HCWs sampled and the different healthcare organizations relating to primary care.

The knowledge of the participants in this study regarding meningococcal disease and its prevention is not fully satisfactory given that, for example, approximately more than three-quarters of the PCPs were aware that MenACWY vaccine is recommended for children in the second year of life and adolescents and 64% of the GPs were aware that MenACWY vaccine is recommended for adolescents. Moreover, the majority of the PCPs and GPs did not rate their knowledge on meningococcal disease and the related vaccination strategy as excellent/very good. These results are lower than those found in a study conducted in the United States where 96% of pediatricians and 70% of GPs answered correctly that MenACWY vaccination is routinely recommended [17]. This lack of knowledge is alarming because especially PCPs have a key role in vaccinations for infants and at-high-risk children, and the correct knowledge on the vaccines’ recommendations are required in order to correctly inform the parents about the risks of meningococcal disease. This is even more important in light of the fact that GPs and PCPs are considered a valid source of information by parents [18,19] as confirmed also by a previous study conducted by some of us on childhood vaccinations, where the self-reported parents’ trust in the pediatricians reached a high value and physicians were the most important source of knowledge on vaccinations as reported by parents [20]. Moreover, the finding that few PCPs and GPs self-rated their knowledge on meningococcal disease and its prevention as excellent/very good could be explained by the fact that they are trained to care for different types of patients and, therefore, they were not fully confident in their knowledge due to the different ages at risk of infection, the different serotypes, and the different recommendations of the available vaccines. Indeed, this also explains the result that a large majority of the study participants reported the need for additional information.

A very positive attitude toward vaccinations against meningococcal disease was found in the participants despite the perception of the risk that their patients could get meningococcal disease was rather low. Indeed, the GPs and PCPs considered vaccinations against meningococcal disease to be very effective and safe. This is an important piece of data because GPs and PCPs are recognized as fundamental healthcare professionals for the correct promotion, counseling, and also administration of vaccinations given that they are widely present throughout the national territory and have in-depth knowledge, compared to other physicians, of their patients who may be more inclined to get vaccinated if recommended by the physicians they trust. Moreover, several factors were significantly associated with the positive attitude regarding the efficacy and safety of vaccination against meningococcal disease, such as older age and a higher level of knowledge regarding the medical conditions that expose patients to the risk of contracting meningococcal disease. This confirmed that positive attitudes toward vaccinations are influenced by correct knowledge.

In this study, a large majority of the GPs and PCPs reported that they recommend meningococcal vaccines to their patients; in particular, very high values were observed among the PCPs for their recommendation to children (98.7%) and adolescents (97.3%) and among the participants for patients at high risk of contracting meningococcal disease for their medical conditions (78.8% of the PCPs and 83.4% of the GPs). Similar results have already been described in a previous study in Turkey where 89.6% of pediatricians recommended meningococcal immunization [14], whereas in a previous investigation in the same geographic area of the present study, even if carried out on a sample of HCWs from different medical areas, only 34.1% of those who provide care to patients with underlying high-risk medical conditions recommended meningococcal vaccinations [11]. Moreover, lower values have been reported in a study conducted in France on GPs where 52% of them reported they always recommend routine MenC vaccination for patients 12 months of age and 33% catch-up vaccination for patients of 2–24 years of age [21], and in a previous investigation in Turkey with 67.9% of family physicians who recommended meningococcal vaccination [12]. Immunization is a fundamental tool especially for preserving the health of fragile patients, and the recommendation of physicians is a very important practice because it has been reported that the lack of recommendation is among the main reasons for low vaccination coverage of the population [22,23,24]. However, it should be underlined that further efforts are needed on the part of healthcare professionals and the organizers of vaccination campaigns in order to improve the coverage because the latest available data show that in Italy the vaccination coverage against meningococcus is still far from the optimal rates [6], although a large majority of physicians reported to recommend vaccinations during their professional practice and to verify the patients’ immunization status. Indeed, several interventions have been found to be effective to increase vaccination rates [25,26,27], including interventions that directly involve physicians. Therefore, it is necessary to take advantage of the fact that GPs and PCPs visit their patients frequently and they can both influence parents’ vaccination choices and have the opportunity to directly administer the vaccinations. Moreover, it is crucial to bring vaccinations as close as possible to susceptible patients through the involvement of other healthcare professionals such as outpatient specialists and those who work in public hospitals, and the role and practices of these healthcare professionals could be the subject of future investigations.

Only a small part of the GPs and PCPs in this study participated in courses/scientific conferences on meningococcal disease and the related vaccination strategies in the last year. These data make it clear that there is still a lot of space to increase the appropriate knowledge and best practices of healthcare professionals on the prevention of meningococcal disease. Indeed, almost all of our sample reported the need for additional information on meningococcal disease and the related vaccinations. It is essential to increase the training of GPs and PCPs on this issue in order to raise their awareness of intercepting and informing parents and especially younger individuals in the most effective way possible on the prevention of meningococcal disease, using the information and communication channels most suited to them as well as, for example, social networks.

Regarding the sources of information, scientific journals were the main source of information on meningococcal vaccinations for the GPs and PCPs. This is an important point as several studies have highlighted that healthcare professionals that used scientific journals as a source of information have more knowledge and more appropriate practices on immunization [28,29,30]. On the other hand, the internet is the second most frequent source of information reported by the participants followed by the mass media, and these data must be worrying because the internet, as well as the mass media, can be a place where false news spreads, especially by unrecognized health authorities or anti-vaccination movements. Even if these aspects mainly concern the general population and the susceptible patients, it cannot be ruled out that healthcare professionals could also be negatively influenced by this information on the Web.

There are potential limitations of this investigation that must be taken into account to correctly interpret the study findings. First, the cross-sectional study design does not allow for an established causal conclusion between the determinants and outcomes of interest. Second, the selection of GPs and PCPs was performed only in Southern Italy (the Campania and Calabria regions) and this may affect the generalizability of the results. Third, the participants’ behaviors regarding the frequency of verifying the patients’ immunization status, the recommendation of the vaccines to different age groups, and the vaccine administration were self-reported, and it is possible that there were desirability and recall biases. 

## 5. Conclusions

In conclusion, the results from this study provide information that can be useful to those involved in health policy to improve prevention programs against meningococcal disease. The findings of the survey highlighted the need for greater engagement by GPs and PCPs in the immunization campaigns in order to increase meningococcal vaccination coverage and to protect especially those younger from meningococcal disease and its serious complications.

## Figures and Tables

**Table 1:** Main sociodemographic and professional characteristics of participants.

Characteristics	N	%
Gender		
Female	219	50.9
Male	211	49.1
Age, years	51.6 ± 0.5 (28–73) *	
Marital status		
Married/cohabitant	393	77.5
Unmarried/separated/divorced/widowed	114	22.5
Having children		
No	166	32.8
Yes	340	67.2
Profession		
General practitioners	340	68.1
Primary care pediatricians	159	31.9
Length of professional practice, years	25.1 ± 12.6 (3–46) *	
Number of hours worked per week	36.7 ± 10.2 (6–80) *	

* Mean ± standard deviation (range). Number for each item may not add up to total number of study population due to missing value.

**Table 2:** Multivariate logistic regression analysis to investigate the variables associated with the outcomes of interest.

Variable	OR	SE	95% CI	*p*
Model 1. GPs and PCPs who considered vaccinations against meningococcal disease to be very effective and safe				
Those who self-rated excellent/very good their knowledge on meningococcal disease	2.83	0.67	1.78–4.51	<0.001
Older age	1.03	0.01	1.01–1.05	0.001
Those who knew the medical conditions that expose patients to a higher risk of contracting meningococcal disease	2.08	0.63	1.14–3.78	0.017
Those who need additional information on meningococcal disease and related vaccinations.	0.53	0.17	0.29–1.02	0.06
Females	1.44	0.33	0.91–2.26	0.117
Scientific journals as the main source of information on meningococcal vaccinations	0.74	0.18	0.45–1.2	0.221
Married/cohabitant	0.75	0.21	0.45–1.27	0.291
Model 2. Physicians who administered vaccines against meningococcal disease to their patients				
Females	1.73	0.21	1.16–3.22	<0.001
General practitioners	0.11	0.03	0.06–0.18	<0.001
Scientific journals as the main source of information on meningococcal vaccinations	1.85	0.58	1.01–3.43	0.05
Those who verified the patients’ immunization status during their professional practice	1.01	0.01	0.99–1.01	0.084
Those who need additional information on meningococcal disease and related vaccinations	1.52	0.58	0.71–3.25	0.284

## Data Availability

The data presented in this study are available upon request from the corresponding author.

## Supplementary Materials

The following supporting information can be downloaded at: https://www.mdpi.com/article/10.3390/vaccines12080889/s1, Supplementary file: Questionnaire.
