# Supplementary material for: Prevention of Meningococcal Disease: Knowledge, Attitudes, and Practices of General Practitioners and Primary Care Pediatricians in South Italy
Source: Vaccines (Basel). 2024 Aug 6;12(8):889. doi: 10.3390/vaccines12080889 (PMC11360620; doi:10.3390/vaccines12080889)
Supplement: Supplementary file 1 [file vaccines-12-00889-s001.zip › vaccines-3100972-supplementary.pdf]

QUESTIONNAIRE

Section A.

This section is designed to gather information about your socio-demographic characteristics.

A1. Gender: □ Male □ Female

A2. How old were you on your last birthday? ______________

A3. What is your marital status? □ Married □ Single (never married) □ Other ___________

A4. Number of children: _______________

A5. Year of graduation: ________________

A6. What is your current occupation? □ General Practitioner □ Primary Care Pediatricians

A7. Number of hours worked per week: _____________________________

A8. Number and age of patients seen in a week: ______

Section B.

This section is designed to gather information about your knowledge about meningococcal disease and related vaccination.

B1. In Italy, the incidence of meningococcal disease is low: □ Yes □ No □ Do not know

B2. Meningococcal disease lethality rate ranges: □ 5-10% □ 10-15% □ 20-25% □ 30-35%

B3. In your opinion, in Italy what are the most frequent serogroups responsible for meningococcal disease?

□ A □ B □ C □ W135 □ X □ Y □ Do not know

B4. In your opinion, which age expose the patients to an higher risk of contracting meningococcal disease? ________

B5. In your opinion, what are the conditions that expose the patients to an higher risk of contracting meningococcal disease?____

B6. According to the most recent recommendations, at what age is the first dose of the quadrivalent meningococcal ACWY (MenACWY) and meningococcal B (MenB) vaccines recommended for newborns? ____

B7. According to the most recent recommendations, at what age is the first dose of the ACWY quadrivalent meningococcal vaccine (MCV4) recommended for newborns?______

B8. In your opinion, which meningococcal vaccine(s) is/are recommended for adolescents?______

B9. How would you consider your knowledge of meningococcal disease?

□ Excellent □ Very Good □ Good □ Poor □ Very Poor

Section C.

This section is designed to gather information about your attitudes about meningococcal disease and related vaccination.

C1. On a scale ranged from 1 to 10, how likely do you think your patient would get a meningococcal disease? (1 unlikely; 10 very likely)

1 2 3 4 5 6 7 8 9 10

C2. On a scale ranged from 1 to 10, how effective do you consider vaccinations against meningococcal disease? (1 not effective; 10 very effective)

1 2 3 4 5 6 7 8 9 10

C3. On a scale ranged from 1 to 10, how safe do you consider vaccinations against meningococcal disease? (1 not safe; 10 very safe)

1 2 3 4 5 6 7 8 9 10

C4. On a scale ranged from 1 to 10, how much you think it is likely that your patient can experience a serious side effect following the administration of anti-meningococcal vaccines? (1 unlikely; 10 very likely)

1 2 3 4 5 6 7 8 9 10

C5. In your opinion, a physician must encourage patients to adhere to vaccinations recommended even if hesitant. □ strongly agree □ agree □ uncertain □ disagree □ strongly disagree

Section D.

This section is designed to gather information about your practice toward the meningococcal vaccine.

D1. During your professional practice, do you periodically verify the immunization status of your patients?

□ Yes □ No

D2. During your professional practice, do you recommend anti-meningococcal vaccinations to the following population groups? □ Children □ Adolescents □ Adults □ Patients at high risk of contracting meningococcal disease for their medical conditions

D3. During your professional practice, do you administer meningococcal vaccines? □ Yes □ No, why?________________________

D4. During your professional practice, how many anti-meningococcal vaccines do you administer per month on average?________

D5. According to your professional experience, how do you consider the acceptability of meningococcal vaccinations by parents or their patients?

D6. In the last 12 months, did you participate in courses/conferences about meningococcal disease and related vaccination strategies?

Section E.

This section is designed to assess the sources from which you acquire information about meningococcal vaccination

E1. What sources of information do you use regarding meningococcal vaccination?

(more than one answer is possible)

□ Scientific journals □ Mass media □ Continuing medical education courses □ Internet

□ Colleagues □ Pofessional associations □ Other _______________

E2. On a scale ranged from 1 to 10, how useful you believe the information received about the prevention of meningococcal disease? (1 not useful; 10 very useful)

1 2 3 4 5 6 7 8 9 10

E3. Do you feel you need additional information on meningococcal disease and related vaccinations? □ No □ Yes
